# Challenging the traditional approach for interpreting genetic variants: Lessons from Fabry disease

**DOI:** 10.1111/cge.14102

**Published:** 2021-12-28

**Authors:** Dominique P. Germain, Thierry Levade, Eric Hachulla, Bertrand Knebelmann, Didier Lacombe, Vanessa Leguy Seguin, Karine Nguyen, Esther Noël, Jean‐Pierre Rabès

**Affiliations:** ^1^ French Referral Centre for Fabry Disease, Division of Medical Genetics AP‐HP University Paris Saclay Garches France; ^2^ Division of Medical Genetics University of Versailles‐Saint‐Quentin‐en‐Yvelines Montigny le Bretonneux France; ^3^ INSERM UMR1037 Cancer Research Center of Toulouse (CRCT) and Paul Sabatier University Toulouse France; ^4^ Clinical Biochemistry Laboratory, Reference Center for Inherited Metabolic Diseases, Federative Institute of Biology University Hospital of Toulouse Toulouse France; ^5^ Department of Internal Medicine and Clinical Immunology Claude Huriez Hospital, University of Lille Lille France; ^6^ Nephrology‐Dialysis Department AP‐HP, Necker Enfants Malades Hospital, University of Paris Paris France; ^7^ Department of Medical Genetics University Hospital of Bordeaux Bordeaux France; ^8^ INSERM U1211, University of Bordeaux Bordeaux France; ^9^ Department of Internal Medicine and Clinical Immunology, François Mitterrand Hospital Dijon University Hospital Dijon France; ^10^ Department of Medical Genetics APHM, Timone Children Hospital Marseille France; ^11^ Department of Internal Medicine Strasbourg University Hospital Strasbourg France; ^12^ Department of Biochemistry and Molecular Genetics Ambroise Paré University Hospital, APHP, Paris‐Saclay University Boulogne‐Billancourt France

**Keywords:** ACMG criteria, experts, Fabry disease, genetic variants, pathogenicity interpretation, variants of unknown significance

## Abstract

Fabry disease (FD) is an X‐linked genetic disease due to pathogenic variants in *GLA*. The phenotype varies depending on the *GLA* variant, alpha‐galactosidase residual activity, patient's age and gender and, for females, X chromosome inactivation. Over 1000 variants have been identified, many through screening protocols more susceptible to disclose non‐pathogenic variants or variants of unknown significance (VUS). This, together with the non‐specificity of some FD symptoms, challenges physicians attempting to interpret *GLA* variants. The traditional way to interpreting pathogenicity is based on a combined approach using allele frequencies, genomic databases, global and disease‐specific clinical databases, and in silico tools proposed by the American College of Medical Genetics and Genomics. Here, a panel of FD specialists convened to study how expertise may compare with the traditional approach. Several *GLA* VUS, highly controversial in the literature (p.Ser126Gly, p.Ala143Thr, p.Asp313Tyr), were re‐analyzed through reviews of patients' charts. The same was done for pathogenic *GLA* variants with some specificities. Our data suggest that input of geneticists and physicians with wide expertise in disease phenotypes, prevalence, inheritance, biomarkers, alleles frequencies, disease‐specific databases, and literature greatly contribute to a more accurate interpretation of the pathogenicity of variants, bringing a significant additional value over the traditional approach.

## Graphical abstract

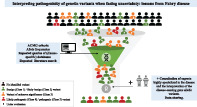

## 1 INTRODUCTION

Fabry disease (FD; OMIM #301500) is a rare X‐linked inborn error of glycosphingolipid metabolism, caused by pathogenic variants in the *GLA* gene (Gene Entrez: 2717; NCBI reference sequence: NM_000169.3; OMIM #300644; Locus Reference Genomic record LRG_672), which encodes the lysosomal enzyme α‐galactosidase A (α‐Gal A, EC 3.2.1.22; Uniprot P06280). Quantitative/functional α‐Gal A deficiency leads to progressive accumulation of its undegraded substrates globotriaosylceramide (Gb_3_) and its deacylated derivative globotriaosylsphingosine (lyso‐Gb_3_), in tissues and body fluids.^1^

FD has two main forms, “classic” and “later‐onset.”^1^, ^2^ “Classic” FD typically manifests during childhood, progressing to life‐threatening renal, cardiac, and/or neurological complications, with hampered quality of life and reduced life expectancy.^1^, ^2^ “Later‐onset” FD typically has cardiac manifestations.^3^, ^4^ FD phenotypes primarily depend upon the disease form, nature of the *GLA* variant, age, gender and residual α‐Gal A activity.^1^

High‐throughput next generation sequencing (NGS)‐based screening programs in high‐risk populations and newborns have identified several novel *GLA* variants. Understanding variant pathogenicity is the key to accurate prevalence estimation, diagnosis, and management of FD. Revised interpretation of *GLA* variants, previously inaptly classified “pathogenic,” has decreased the estimates of FD prevalence (Table 1).^5^, ^6^, ^7^, ^8^

**TABLE 1:** Revised prevalence of Fabry disease in various screening populations after re‐interpretation of pathogenicity

Patient population	Previous prevalence^5^, ^6^	Revised prevalence^7^, ^8^	Absolute prevalence^7^, ^8^
**Newborn males** ^5^, ^7^	0.03%^5^	0.014%^7^	1 in 6883^7^
**Adult males** ^6^, ^8^			
Hemodialysis	0.33%	0.21%	1 in 476
Renal transplant	0.38%	0.25%	1 in 400
Hypertrophic cardiomyopathy	2.67%	0.94%	1 in 106
Cryptogenic strokes	4.23%	0.13%	1 in 769
**Adult females** ^6^, ^8^			
Hemodialysis	0.10%	0.15%	1 in 667
Renal transplant	0%	0%	
Hypertrophic cardiomyopathy	2.8%	0.90%	1 in 111
Cryptogenic strokes	2.13%	0.14%	1 in 714

Over 1000 *GLA* variants have been reported,^9^, ^10^, ^11^, ^12^ most of them private, including some variants of unknown significance (VUS).^13^ Furthermore, the non‐specificity of FD symptoms presents a challenge for physicians attempting to interpret the clinical relevance of a VUS.

A panel of French physicians involved in the diagnosis and management of FD (clinical and molecular geneticists, nephrologists, and internal medicine specialists), convened to discuss the diagnostic challenges posed by *GLA* VUS. This paper illustrates the challenges in interpretating pathogenicity of *GLA* VUS through real‐life cases, highlights the input of highly specialized experts and provide practical recommendations for accurate diagnosis of FD.

## 2 TERMINOLOGY

In FD, the term “variant” had been historically used to describe the two main forms/phenotypes, creating confusion between genetic and clinical levels when the Human Genome Variation Society (HGVS) advised to replace “mutation” by the term “variant.”^14^ We therefore recommend keeping the term “variant” to describe *GLA* alleles and using “form” or “phenotype” for clinical description of the disease (i.e., classic or later‐onset form/phenotype of FD). The terms “mutation” and “polymorphism” should be avoided, due to implied pathogenicity and benignity, respectively. Variants are categorized as benign (class 1), likely benign (class 2), of uncertain significance (VUS, class 3), likely pathogenic (class 4), or pathogenic (class 5).^13^

## 3 PHENOTYPES AND UNDERLYING BIOCHEMICAL MECHANISMS

### 3.1 Clinical phenotypes

**FIGURE 1:**
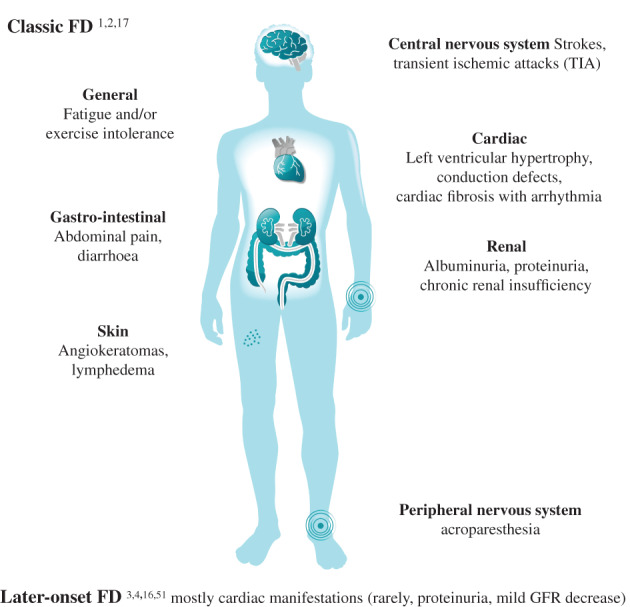
Main clinical manifestations of Fabry disease (FD)

The “classic” phenotype typically manifests during childhood,^15^ progressing in adulthood to life‐threatening complications, hampered quality of life and reduced life expectancy (Figure 1).^1^, ^2^ The later‐onset FD phenotype mostly exhibits cardiac manifestations, which appear later in life.^1^, ^3^, ^4^, ^16^

Absent/very low residual α‐Gal A activity is associated with the more severe classic phenotype, earlier onset of symptoms, and multi‐organ involvement.^1^, ^17^, ^18^ However, as many genetic diseases, FD has a highly variable phenotypic expressivity.

### 3.2 Biochemical phenotypes

Alpha‐Gal A activity can be measured in leukocytes, plasma, or dried blood spot (DBS) cards. Reference values vary widely depending on pre‐analytical and technical conditions. The activity of another lysosomal enzyme, e.g., β‐galactosidase or hexosaminidase, should be simultaneously determined for quality control. Alpha‐Gal A deficiency is expressed in micromole/L/h.^19^, ^20^ In males, substrate accumulation starts once α‐Gal A activity drops below 20%–25% of normal levels.^1^ In severe deficiency, α‐Gal A levels are <1%–3% of mean control values. Although low α‐Gal A activity in females is a good indicator of pathogenicity,^21^ its diagnostic value in females is diminished due to normal α‐Gal A activity in several heterozygous females with confirmed FD, depending on X‐chromosome inactivation (XCI).^21^

**Globotriaosylsphingosine or lyso‐Gb**_**3**_ (α‐d‐galactopyranosyl‐(1,4)‐β‐d‐galactopyranosyl‐(1,4)‐β‐d‐glucopyranosyl‐(1,1)‐(2S,3R,4E)‐2‐amino‐octadec‐4‐ene‐1,3‐diol) is the deacylated form of Gb_3_. Increased plasma lyso‐Gb_3_ levels in male FD patients were first reported in 2008.^22^ This amphipathic lipid, found at high concentration in plasma of male FD patients, has been attributed to the action of acid ceramidase on Gb_3_. Lyso‐Gb_3_ has progressively superseded Gb_3_ as the main biomarker for diagnosing and monitoring FD.

#### 3.2.1 Is lyso‐Gb_3_ causally involved in the pathogenesis of FD?

Increasing evidence implicates lyso‐Gb_3_ in the pathogenesis of FD, possibly through inflammation.^23^, ^24^ The addition of lyso‐Gb_3_ to cultured smooth muscle cells induces cell proliferation, suggesting its role in the vascular remodeling characteristic of FD.^20^ The inhibitory effect of lyso‐Gb_3_ on endothelial nitric oxide synthase may also contribute to vascular dysfunction in FD patients.^25^ Lyso‐Gb_3_ may induce glomerular damage through activation of Notch1 signaling and TGF‐β1‐mediated production of extracellular matrix by podocytes.^26^ Recent studies suggest an activation of innate immunity and possibly of adaptive immunity by lyso‐Gb_3_ in target tissues including myocardium and kidney. In a recently proposed model of cardiac FD staging, myocardial inflammation can also precede the effects of Gb_3_ storage on cardiac wall thickness and function.^27^

#### 3.2.2 Can lyso‐Gb_3_ be used as a biomarker in FD?

Plasma lyso‐Gb_3_ levels are dramatically increased in male patients with classic FD (up to 100‐fold control values).^22^, ^28^, ^29^, ^30^, ^31^, ^32^ Concentrations in males with a mild phenotype and in heterozygous classic females are lower, but nonetheless abnormal.^28^, ^29^, ^30^, ^31^, ^32^ In male FD patients, lyso‐Gb_3_ levels are higher in those with frameshift and nonsense variants than in those with missense variants.^33^ Plasma lyso‐Gb_3_ can reliably distinguish classically affected male and female FD patients from individuals without FD.^30^ Normal lyso‐Gb_3_ levels are insufficient to exclude FD in women, but are highly unlikely in male FD patients. Interestingly, several studies have demonstrated normal plasma lyso‐Gb_3_ levels in individuals carrying benign *GLA* variants.^32^, ^34^, ^35^ A moderate increase in plasma lyso‐Gb_3_ levels was reported to indicate FD in patients with an uncertain diagnosis of FD carrying a VUS, while normal plasma lyso‐Gb_3_ levels were observed in individuals with negative tissue biopsies.^30^

Multiple studies have investigated the association between plasma lyso‐Gb_3_ levels and clinical FD manifestations. In both male and female FD patients, a correlation between plasma lyso‐Gb_3_ exposure and disease severity was reported.^29^ Plasma lyso‐Gb_3_ concentration was identified as an independent risk factor for cerebrovascular white matter lesions in male patients and left ventricular hypertrophy in female patients.^29^ A study of patients with genetic variants associated with classic FD found a correlation between lyso‐Gb_3_ concentrations and left ventricular mass index, but not kidney function.^36^ In later‐onset FD patients carrying the c.644A>G; p.(Asn215Ser)/p.N215S variant, lyso‐Gb_3_ levels correlated with left ventricular mass, glomerular filtration rate, and overall disease severity.^16^ Finally, a correlation between highly elevated plasma lyso‐Gb_3_ levels and severe clinical events has been recently reported.^37^ Urinary lyso‐Gb_3_ levels are also increased in FD patients, and correlate with Gb_3_ levels.^38^ Like urinary Gb_3_ concentration, total urinary concentration of lyso‐Gb_3_ and its structural analogs represent a specific diagnostic biomarker, and is elevated in both classical and non‐classical FD patients.^39^

#### 3.2.3 Does lyso‐Gb3 have a prognostic value in therapeutic drugs monitoring in FD patients?

In hemizygous FD patients, enzyme replacement therapy (ERT) markedly reduces (though does not always normalize) plasma lyso‐Gb_3_ levels within 3 months, thereafter, remaining relatively stable.^40^, ^41^ Similar reduction or stabilization of lyso‐Gb_3_ levels was reported for female FD patients.^41^ Later treatment initiation results in less effective lyso‐Gb_3_ clearance. In male patients with classic FD, lyso‐Gb_3_ levels a year after ERT initiation were lower in patients who began ERT before age 25, versus those who started later in life.^42^ An early initiation of ERT in two yet clinically asymptomatic children with classic FD completely normalized lyso‐Gb_3_.^43^ In FD patients, treatment with oral chaperone migalastat for 1–2 years led to reduced, but not normalized, plasma lyso‐Gb_3_ levels.^44^, ^45^ Regular monitoring of biochemical response to chaperone therapy (e.g., plasma lyso‐Gb_3_ and α‐Gal A activity) is crucial to adjust treatment approach, and patient clinical control at least every 6–12 months.^46^ In summary, state‐of‐the‐art knowledge indicates that lyso‐Gb_3_ is a useful biomarker both for diagnosing and monitoring FD patients.

### 3.3 Additional factors influencing phenotype in Fabry disease

Multiple factors may contribute to the phenotypic heterogeneity associated with a given *GLA* variant.^47^ Theoretically, modifier genes may alter the expression of *GLA* or another disease‐causing gene influencing FD phenotype.^48^ As in other genetic diseases, environmental factors (e.g., hypertension, smoking) may also contribute to phenotypic variability.

The clinical presentation is more variable in heterozygous females than hemizygous males, likely due to epigenetics.^1^, ^21^, ^49^ The inactivation of one of the two X‐chromosomes is generally random in females, though some females have preferential inactivation of the same chromosome in >80% of cells (skewed XCI), affecting the clinical phenotype and prognosis of FD.^21^ Epigenetic regulation, therefore, largely explains the variable expressivity observed for a same *GLA* variant in female FD patients.

## 4 *GLA* ALLELIC HETEROGENEITY: DEALING WITH UNCERTAINTY

**FIGURE 2:**
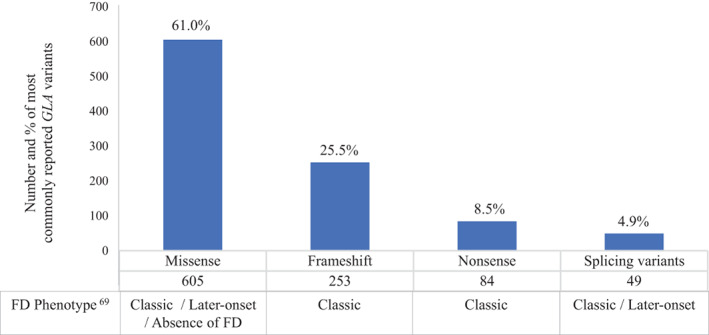
Most commonly reported types of GLA variants in the Human Gene Mutation Database (HGMD) and related Fabry disease (FD) phenotypes in hemizygous patients

Diagnosing FD is often challenging due to *GLA* high allelic heterogeneity (Figure 2), possible absence of male cases in the family and consequent enzymatic values and non‐specificity of (early‐onset) symptoms.

Classic FD with absent or negligible α‐Gal A activity is associated with several nonsense *GLA* pathogenic variants including but not limited to CpG hotspots [e.g. c.658C>T; p.(Arg220*), c.679C>T; p.(Arg227*), c.901C>T; p.(Arg301*)] and missense variants impacting the active site or key residues of the enzyme [e.g. c.509A>G; p.(Asp170Gly), c.508G>C; p.(Asp170His), c.508G>A; p.(Asp170Asn), c.509A>T; p.(Asp170Val)].^9^, ^11^, ^12^ Other missense or splice‐site *GLA* variants may, in contrast, be associated with later‐onset FD and a residual enzyme activity between 3% and 25% of normal in male patients, including c.337T>C; p.(Phe113Leu)/p.(F113L)^50^ and c.644A>G; p.(Asn215Ser)/p.(N215S),^51^ both prevalent in Caucasian populations, and c.639+919G>A (also known as c.640‐801G>A or IVS4+919G>A), the most prevalent variant in Chinese populations.^17^, ^52^

Some *GLA* variants have been classified as VUS, partly because many of them are private to one or few families and because ACMG‐AMP classification is difficult to implement in routine practice. Interpreting variants is particularly complex in the later‐onset phenotype, which may mimic isolated HCM and lack other FD features.^47^ The pathogenicity of several missense *GLA* variants has been extensively discussed in the literature. However, for some of them it remains unclear, requiring insights from the experts for an accurate interpretation.

Hereafter, eight cases illustrating the challenges in interpretation of *GLA* variants' pathogenicity, and the insights from field experts, are presented. Written informed consent was obtained for all cases.

### 4.1 *GLA* variants not resulting in FD diagnosis

#### 4.1.1 c.427G>A; p.(Ala143Thr)/p.(A143T)

A 41‐year‐old man was referred for transient ischemic attack (TIA) with dysarthria and central paralysis of the left VII cranial nerve. Brain magnetic resonance imaging (MRI) revealed sequelae of a recent minor stroke and multifocal punctate white matter lesions, mainly in the periventricular area. Medical history included hypertension and tobacco use. Classic FD signs (angiokeratoma or *cornea verticillata*), renal or ear involvement were all absent. Holter‐ECG (24‐h) was normal. Cardiac MRI revealed a normal left ventricular mass (81 g/m^2^) and gadolinium late‐enhancement showed subendocardial (not intramural) fibrosis of inferolateral, anterolateral, and septal walls, indicative of ischemia rather than FD. Significant obstructive coronary disease was absent. Plasma α‐Gal A levels were slightly reduced (6.7 μkat/kg) with normal plasma lyso‐Gb_3_ (0.3 ng/ml; normal values <1.8 ng/ml) and urinary Gb_3_. Molecular genetic analyses identified a hemizygous missense *GLA* variant c.427G>A; p.(Ala143Thr).

The patient was initially diagnosed with FD and disease‐specific treatment was initiated. He remained stable for >7 years. Family screening and segregation analysis revealed his 68‐year‐old mother and 31‐year‐old brother as asymptomatic p.(Ala143Thr) carriers, suggesting the non‐pathogenicity of this *GLA* variant. The patient's brother had only slightly decreased α‐Gal A activity, and neither cardiovascular nor FD signs or symptoms.

Considering the low specificity of a single disease symptom, high residual enzyme activity and recent literature strongly questioning the pathogenicity of the p.(Ala143Thr) variant, diagnosis of FD in this patient was reconsidered and finally ruled out/excluded. Other etiologies for cardiac fibrosis are being investigated.

A 44‐year‐old female patient with a history of pain due to polyarthralgia and rachialgia, and ischemic stroke of unknown etiology consulted a rheumatology department. Molecular *GLA* analysis identified the p.(Ala143Thr) variant. Investigation revealed arthritis of the second and third metacarpophalangeal joints, but no signs of FD. Alpha‐Gal A activity and plasma lyso‐Gb_3_ (0.3 ng/ml; normal values <1.8 ng/ml) were normal. Family screening identified p.(Ala143Thr) in the patient's 15‐ and 21‐year‐old sons and 35‐year‐old sister. Neither of her sons had FD signs/symptoms and therefore refused further investigations including enzymatic assay, which would have been diagnostic. Extensive investigations in the patient's sister, who had multiple sclerosis, did not reveal any sign of FD. The index case was subsequently diagnosed with spondylarthritis, while FD was excluded.

A 25‐year‐old male patient with moderate HCM was referred for further analyses. Septal wall thickness was 17 mm. Ventricular obstruction, dilation and rhythm anomalies were absent. Left ventricular ejection fraction was 60%. There were no features of FD. Family history revealed that patient's maternal uncle, with the p.(Ala143Thr) variant, had been considered 12 years earlier with a possible diagnosis of FD, which was subsequently reconsidered and excluded on the basis of α‐Gal A activity only reduced to 35% (in a male) and normal urinary Gb_3_ (1.8 nmol/mmol creatinine; normal values <8 nmol/mmol creatinine). Extensive analysis of a panel of sarcomeric genes involved in hereditary cardiomyopathies revealed c.6327_6332del; p.(2109_2111del), a pathogenic variant in the *FLNC* gene (known to cause sarcomeric autosomal dominant cardiomyopathies). This *FLNC* variant was inherited from his 57‐year‐old mother, who will be further investigated.

The missense p.(Ala143Thr) variant was first described in 1997^53^ in an asymptomatic 2‐month‐old infant with low enzymatic activity but no family history of FD. Software tools predict p.(Ala143Thr) to impact the biological function of α‐Gal A. Databases associate p.(Ala143Thr) to FD, except dbFGP (International Fabry Disease Genotype–Phenotype Database) and LOVD (Leiden Open Variation Database) (Table 3). The variant p.(Ala143Thr) has long been considered pathogenic and disease‐modifying treatments were unnecessarily indicated.^8^ As in the case of the 41‐year‐old male patient described above, various studies of patients hemizygotes for p.(Ala143Thr) with stroke/TIA demonstrated only slightly decreased α‐Gal A activity when compared to other *GLA* missense variants. Those patients had normal lyso‐Gb_3_ levels, no typical FD symptoms, Gb_3_ negative biopsies and generally an absence of stroke/TIA in other family members carrying p.(Ala143Thr).^35^, ^54^ Alpha‐Gal A residual activity in males with p.(Ala143Thr) ranges between 25% and 72% of normal,^30^ in line with 46% reported for the 41‐year‐old male and 35% for the 72‐year‐old male described above. This expert panel considers p.(Ala143Thr) to be likely benign. An important evidence is that the prevalence of p.(Ala143Thr) in the non‐Finish European population (0.095%, Table 3) is higher than the prevalence of FD itself. Interestingly, in individuals with p.(Ala143Thr) identification in high‐risk population screenings, the only association with FD is the event that motivated the screening, in favor of a result driven by inclusion bias. For example, TIA/stroke was found in p.(Ala143Thr) patients, when screening population with TIA/stroke of unknown cause, but no higher frequency of end‐stage renal disease was found in that population; the same observation applies for other entry points that motivated screening.^35^, ^54^ Likewise, due to its not so rare prevalence in the general population (0.051% and 0.13% in the Europeans and Americans, respectively) (https://evs.gs.washington.edu/), p.(Ala143Thr) is the paradigm of a coincidental finding while exploring a variety of genetic diseases, such as MYH‐associated polyposis, achromatopsia, hereditary cancer‐predisposing syndrome, and renal dysplasia, where it should not be considered causal (similarly to the aforementioned strokes/TIA).^55^

The high residual enzyme activity associated with p.(Ala143Thr) is in favor of a pseudo‐deficiency rather than a disease‐causing allele per se.^35^, ^56^ Of note, the dbFGP database, which includes inputs from FD experts and researchers, clearly states p.(Ala143Thr) as benign (Table 3).

#### 4.1.2 c.937G>T; p.(Asp313Tyr)/p.(D313Y)

A 67‐year‐old female had undergone chronic hemodialysis since age 26, due to biopsy‐confirmed membranoproliferative glomerulonephritis with unclear etiology. DBS analysis demonstrated reduced α‐Gal A activity (0.99 μmol/l/h; normal: 1.5–2 μmol/l/h) and normal lyso‐Gb_3_ (1.18 ng/ml; normal: 0.0–3.5 ng/ml). The *GLA* variant c.937G>T; p.(Asp313Tyr) was identified, but an expert geneticist (D.P.G.) advised to exclude FD, since there was no other clinical sign of FD or relevant family history, apart from slightly decreased α‐Gal A activity (57% of normal). Renal biopsy was not suggestive of FD.

A 50‐year‐old male consulted due to a family history of HCM and *GLA* variant p.(Asp313Tyr)/Tyr313, also identified in his two sisters affected with familial cardiomyopathy and his asymptomatic mother. Notably, the patient's father had died of a cardiac arrest, which raises suspicion of an autosomal dominant transmission. Extensive investigations did not reveal any FD feature. In contrast, additional molecular studies using a panel of five sarcomeric genes identified a pathogenic variant in the *TNNT2* troponin T gene. This variant was likely responsible for the familial cardiomyopathy, inherited from the patient's father whereas the p.(Asp313Tyr) *GLA* variant inherited from patient's mother was coincidental.

The missense p.(Asp313Tyr) variant was historically considered pathogenic for a while.^57^ It was first described in 1993 in a classic FD male patient with only partial sequencing of the *GLA* gene, though clinical description was not provided. However, another group subsequently published a case with the additional presence of another *GLA* variant [c.1232G>A; p.(Gly411Asp)], creating an uncertainty regarding pathogenicity of p.(Asp313Tyr).^58^ Functional studies showed that p.(Asp313Tyr) is a sequence variant associated with 75% of normal α‐Gal A activity (pseudo‐deficiency).^58^ Similarly, cells transfected with p.(Asp313Tyr) showed even higher residual α‐Gal A levels (59%) than cells transfected with other non‐pathogenic *GLA* variants, that is, c.352C>T; p.(Arg118Cys)/p.(R118C) (24.5%),^59^ and c.196G>C; p.(Glu66Gln)/(p.(E66Q) (47.6%).^35^, ^60^, ^61^ While in vitro prediction tools give conflicting predictions for this variant (Table 3), p.(Asp313Tyr) prevalence in the non‐Finnish European population is 0.45% in the GnomAD database, much higher than the global FD prevalence. Such high allelic frequency supports the classification of p.(Asp313Tyr) as benign. Three major databases (dbFGP, ClinVar and LOVD) agree on classifying p.(Asp313Tyr) as benign/likely benign (Table 3). Several recent reports on p.(Asp313Tyr) demonstrated clinical and biochemical absence of FD features and concluded that p.(Asp313Tyr) per se does not cause FD.^35^, ^62^

#### 4.1.3 c.376A>G; p.(Ser126Gly)/p.(S126G)

A 45‐year‐old Italian male was referred to the French Referral Center for Fabry disease for a suspicion of familial FD in association to the p.(Ser126Gly) *GLA* variant. His sister, an asymptomatic p.(Ser126Gly) *GLA* carrier, was already considered for FD‐specific treatment in Italy. Extensive investigations did not reveal any sign or symptom of FD; α‐Gal A levels were 57% of normal in two independent assays. Brain MRI, glomerular filtration rate and lyso‐Gb_3_ were normal and FD was consequently excluded.

p.(Ser126Gly) has been found in 74 cases (exomes frequency of 0.033%, *n* = 60; genomes frequency of 0.063%, *n* = 14) in GnomAD, above the global frequency of FD (Table 3). While several publications claimed p.(Ser126Gly) to be pathogenic when associated with a given haplotype (though no convincing clinical evidence was provided),^63^ this expert opinion authors considers p.(Ser126Gly) as likely benign, in line with all major databases which do not associate p.(Ser126Gly) with FD (Table 3).

### 4.2 Novel *GLA* variants resulting in FD diagnosis

#### 4.2.1 c.931dupC; p.(Leu311Profs*4)

A 48‐year‐old Algerian female was referred for evaluation of isolated HCM. The patient reported acroparesthesia during childhood. NGS sequencing of genes involved in hereditary cardiomyopathies identified a novel, previously undescribed frameshift *GLA* variant, c.931dupC; p.(Leu311Profs*4), which was categorized as pathogenic. Patient had elevated lyso‐Gb_3_ (10.8 ng/ml, normal: <1.8 ng/ml). Investigations found no renal/neurological involvement, angiokeratoma, or *cornea verticillata*. Her two sisters, (both with HCM) and rest of family were unavailable for further evaluation. FD was confirmed and disease‐specific treatment initiated. The frameshift associated with this variant was predicted to result in classic FD even though patient's symptoms were mostly limited to the heart, illustrating the phenotypic variability of many genetic diseases.

#### 4.2.2 c.1281_1282insCTTA; p.(Leu429Ilefs*22)

A 54‐year‐old male was hospitalized for syncope, due to complete atrioventricular block; a dual chamber pacemaker was implanted. He had no FD relevant medical history (except for clustered angiokeratomas). His mother had died from sudden cardiac arrest at age 69. Investigations revealed markedly reduced leukocyte α‐Gal A activity (1 nmol/h/mg; normal: 16–39 nmol/h/mg) and elevated plasma lyso‐Gb_3_ levels (10.1 ng/ml; normal: <5 ng/ml). Fabry disease diagnosis was confirmed, and disease‐specific treatment initiated, together with close clinical and biochemical follow‐up. Additional tests showed microalbuminuria, presence of T2/FLAIR signal alterations in the periventricular white matter on brain MRI, normal audiogram, and absence of *cornea verticillata*. Family screening is ongoing for his two sisters (aged 57 and 59), his brother (aged 42), and his two daughters (aged 26 and 27).

*GLA* genotyping revealed a novel hemizygous 4‐base insertion (c.1281_1282insCTTA) predicted to preserve the entire coding sequence of α‐galactosidase A, with the exception of a conservative change of the last amino‐acid (leucine to isoleucine) at codon 429, together with the addition of a 22‐amino‐acid tail. A frameshift variant is theoretically predicted to cause a severe full‐blown classic phenotype in a 54‐year‐old male patient, which was not the case in this hemizygote with predominantly cardiac symptoms. This may be due to the unique nature of the genetic variant preserving the coding sequence until the last codon of the protein.

### 4.3 Databases and in‐silico prediction softwares

Genetic databases and in silico prediction softwares are useful resources (Table 2), but physicians with limited FD experience may face conflicting results of pathogenicity depending on the database queried and the time of the query. For example, the classification of the p.(Ala143Thr) variant was changed from “likely pathogenic” (on December 5th, 2019) to VUS (on January 20th, 2020) in Varsome (https://varsome.com) with the addition of “BS1” (**B**enign **S**trong criterion number 1, AF higher than expected for the disorder). It changed again from “VUS” to “pathogenic” on August 3rd, 2020, with the addition of the “PS3” (Pathogenic Strong criterion number 3), as a Varsome user reported this variant as “likely pathogenic” in a paper supported by a functional expression study, with additional removal of both criteria BS1 and BS2 criteria. Furthermore, p.(Ala143Thr) is classified as benign in dbFGP, with conflicting results in other databases (ClinVar, LOVD, the Japanese Fabry Database) (Table 3).

**TABLE 2:** Available databases and in silico prediction softwares

Name	Description
**Databases**	
Genome Aggregation Database (gnomAD) https://gnomad.broadinstitute.org/	Includes aggregated exome and genome sequencing data. Useful to compare the frequency of a variant in the general population or in an ethnic group against the disease prevalence or against the frequency of the most common pathogenic variant. A *GLA* variant is not considered disease‐causing if its frequency is higher than FD prevalence or the reported most frequent variant (Popmax Filtering AF).
ClinVar https://www.ncbi.nlm.nih.gov/clinvar	Collects evidence‐supported interpretations of clinical significance of variants for FD submitted by clinical testing laboratories, researchers, other databases.
Leiden Open (source) Variation Database (LOVD) https://www.lovd.nl/	Displays gene variants.
OMIM® (Online Mendelian Inheritance in Man) https://omim.org/	Contains information on all known Mendelian disorders and over 15,000 genes, focusing on the relationship between phenotype and genotype.
International Fabry Disease Genotype‐Phenotype Database (dbFGP) http://dbfgp.org/dbFgp/fabry	Combines data from databases such as the Human Gene Mutation Database (HGMD) and The Japanese Fabry Database, diagnostic and clinical evaluations of patients with data from peer reviewed publications and input from expert FD researchers and care teams. The database provides information of the associated phenotype for a given variant.
The Japanese Fabry Database http://fabry‐database.org/mutants/	Created by Meiji Pharmaceutical University and led by H. Sakuraba, this database lists *GLA* variants and the reported clinical phenotypes with references.
**In silico prediction softwares**	
VarSome https://varsome.com/	Annotation tool that classifies the genetic variant according to ACMG criteria. The verdict arises from a vast quantity of accurate curated data such as coding impact of the variant, in vitro functional studies, allele frequency, previous publications and other databases analyses.
PolyPhen‐2 http://genetics.bwh.harvard.edu/pph2/	A tool which predicts whether an amino acid substitution or indel has an impact on the biological function of a protein.
Provean (Protein Variation Effect Analyzer) http://provean.jcvi.org/index.php	A tool which predicts whether an amino acid substitution or indel has an impact on the biological function of a protein.
SIFT https://sift.bii.a‐star.edu.sg/	A tool which predicts whether an amino acid substitution or indel has an impact on the biological function of a protein.
Mutation taster http://www.mutationtaster.org/	A tool which predicts whether an amino acid substitution, insertion or deletion has an impact on the biological function of a protein.

**TABLE 3:** Characteristics of common controversial *GLA* variants according to genetic databases and in silico prediction softwares

	c.427G>A; p.(Ala143Thr) / p.(A143T) / Thr143	c.937G>T; p.(Asp313Tyr) / p.(D313Y) / Tyr313	c.196G>C; p.(Glu66Gln) / p.(E66Q) / Gln66	c.352C>T; p.(Arg118Cys) / p.(R118C) / Cys118	c.376A>G; p.(Ser126Gly) / p.(S126G) / Gly126
**GnomAD v2.1.1**					
AF (%) in exomes, genomes (total)	0.055, 0.018 (0.051)	0.30, 0.31 (0.30)	0.012, 0.0045 (0.011)	0.022, 0.032 (0.023)	0.033, 0.063 (0.036)
Highest AF (%) by population	0.095 in European (non‐Finnish)	0.69 in Ashkenazi Jewish 0.45 in European (non‐Finnish)	0.15 in East Asian	0.044 in European (non‐Finnish)	0.074 in European (non‐Finnish)
**Pathogenicity according to FD‐specific databases**					
dbFGP	Benign	Benign	Benign	Benign	Likely benign
The Japanese Fabry Database	LO [5]; classic [4]; B [3]; VUS [1]; np [8]	Classic [5]; B [2]; LO [1]; np [9]	B [5]; classic [5]; LO [3]; np [3]	LO [2]; np [5]	LO [1]; np [6]
**Pathogenicity according to general databases**					
ClinVar	VUS [10]; LP [4]; P [2]	LB [13]; VUS [3]; B [2]	VUS [4]; LB [2]	VUS [12]; LP [2]; LB [1]	LB [6]; VUS [4]; B [1]
LOVD	LB [2]; VUS [1]	LB [3]; B [2]; VUS [1]	np	VUS [3]; P [1]	2 LB [2]; VUS [1]
OMIM	FD	VUS (recently reclassified)	Functional polymorphism and not disease causing	not provided	not provided
ACMG classification according to VarSome (date of query)	**LP** (2019‐12‐05) **VUS** because highest ethnic frequency = 0.10% (2020/01/20) **P** because a user has reported this variant is classified LP in one article (Spada et al.^5^) and that is confirmed by a functional study (2020‐08‐03)	**VUS** (2019‐12‐05) **B** because highest ethnic frequency = 0.69% (2020‐01‐20) **LP** because alternative variant (Asp313Asn) is classified P by UniProt Variants (and confirmed using ACMG) (2020‐08‐04)	**VUS** (2019‐12‐05) **B** because highest ethnic frequency = 0.15% (2020‐01‐20) **LP** because highest ethnic frequency no longer takes into account again (2020‐08‐04)	**LB** (2019‐12‐05) **B** (2020‐01‐20) **LB** (2020‐08‐04)	**VUS** (2019‐12‐05) **B** because highest ethnic frequency = 0.074% (2020‐01‐20) **VUS** because highest ethnic frequency no longer taken into account (2020‐08‐04)
Polyphen‐2	Probably damaging (1)	Probably damaging (0.996)	Probably damaging (0.996)	Probably damaging (0.993)	Benign (0.043)
Provean	Deleterious (−3.119)	Deleterious (−3.183)	Deleterious (−2.754)	Deleterious (−4.667)	Deleterious (−2.823)
SIFT	Damaging (0.004)	Damaging (0.001)	Damaging (0.002)	Damaging (0.001)	Tolerated (0.060)
Mutation taster	Disease causing	Polymorphism	Disease causing	Polymorphism	Disease causing

*Note*: Last accessed 2020‐08‐04. [ ]: The number of times referenced.

Abbreviations: AF, allele frequency; B, benign; dbFGP, International Fabry Disease Genotype–Phenotype Database; FD, Fabry disease; gnomAD, Genome Aggregation Database; LB, likely benign; LOVD, Leiden Open (source) Variation Database; LP, likely pathogenic; np, not provided; P, pathogenic; VUS, variant of unknown significance.

While in silico prediction softwares (e.g., PolyPhen‐2, PROVEAN, SIFT, Mutation Taster) can help in determining the pathogenicity of missense variants, their partial reliability is well‐known as confirmed with/for the five most common (likely) benign *GLA* variants (Table 3).

With respect to databases, those specific for FD and curated by FD experts, appear to be most valuable for classifying *GLA* variant pathogenicity (Table 2).

### 4.4 Literature search

A thorough literature search can also help to determine the pathogenicity of allelic variants. In a recent paper, a group of FD experts, including senior medical geneticists, re‐examined information (clinical, biochemical, and histopathological) from 22 individuals with the p.(Arg118Cys) *GLA* variant. This variant had been originally considered pathogenic, based on poorly documented cases, low AF and prediction of the consequence of amino acid substitution (Table 3).^59^ Careful reassessment of patients and their families ruled out FD, identifying alternative causes for renal failure (e.g., HIV‐associated nephropathy, old age) and stroke (multiple major cardiovascular risk factors).^59^ In agreement, dbFGP and ACMG criteria categorize p.(Arg118Cys) as benign and likely benign, respectively (Table 3).

While the p.(Glu66Gln) *GLA* variant was first suggested to cause FD over 20 years ago,^64^ re‐analysis by Japanese^60^ and Korean^65^ expert groups have subsequently excluded its pathogenicity. Several studies have reported that patients with p.(Glu66Gln) had α‐Gal A levels about 56% (24%–65%) of normal, normal lyso‐Gb_3_ and normal tissue biopsies. Frequency of this variant in Asian populations may reach 0.83% in Japan and 1% in South Korea, which is much higher than FD global prevalence.^60^, ^65^, ^66^, ^67^ It should be noted that literature search yields conflicting results when interpreting p.(Glu66Gly) pathogenicity with data from recognized expert groups in favor of benignity of this *GLA* variant.^60^, ^65^, ^67^

## 5 LESSONS FROM FD FOR MORE ACCURATE VARIANT INTERPRETATION

**FIGURE 3:**
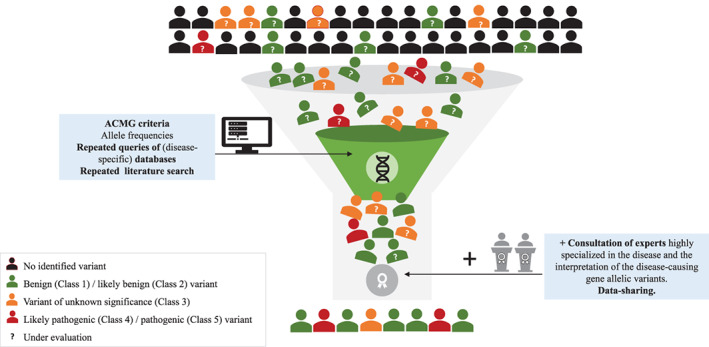
Interpreting pathogenicity of genetic variants when facing uncertainty: lessons from Fabry disease (FD)

Distinguishing disease‐causing variants from benign bystanders is probably one of the biggest challenges in contemporary clinical genetics. Below, the authors provide practical recommendations for accurate diagnosis of FD (Table 4, Figure 3), which may be extended to some other genetic conditions.

**TABLE 4:** Practical recommendations for a more accurate diagnosis of Fabry disease

Combine insights from disease specific databases and in vitro prediction tools with expert clinical opinion, defining the relative weight of each ACMG criterion. Consider the genetic variant as probably benign if its allele total or subpopulation frequency is: Higher than the overall prevalence of FD (0.0125%), Higher than the frequency of the most common pathogenic allele. Perform segregation analysis when additional information is required. Review the literature and databases periodically to check whether the variant has been reclassified.

### 5.1 Insights from disease specific biomarkers

In FD and other genetic diseases of metabolism, surrogate biomarkers are available, which, when properly used, can optimize interpretation of the clinical relevance of a genetic variant. In male patients, α‐Gal A levels are key to confirm or exclude pathogenicity of a variant. In both genders, plasma lyso‐Gb_3_ levels may help in the interpretation, although the possibility of normal lyso‐Gb_3_ levels in heterozygotes affected with the later‐onset phenotype should be considered.

In the case of FD, where a “pharmacogenetic” specific treatment exists, it should be noted that “amenability” of a given *GLA* variant does not necessarily imply pathogenicity, since the available amenability table includes proven non‐pathogenic variants such as p.(Arg118Cys), p.(Ala143Thr) and p.(Asp313Tyr) (www.galafoldamenabilitytable.com, last accessed on November 24th, 2021).

### 5.2 Insights from disease knowledge and expert recommendations to weight ACMG criteria

*GLA* sequencing is conducted in two contexts: clinical suspicion of FD, or screening at‐risk populations (i.e., HCM, end‐stage renal disease, cryptogenic stroke/TIA). Screening of large at‐risk populations will therefore disclose *GLA* VUS with relatively high prevalence. In patients with *GLA* VUS and a non‐specific phenotype, other common genetic causes should be systematically searched (e.g., sarcomeric genes in HCM as illustrated with both aforementioned cases). Erroneous classification of benign variants as pathogenic delays correct diagnosis and can prompt expensive unnecessary treatments. In contrast, detection of a likely benign *GLA* variant in a patient with very strong clinical suspicion of FD, should prompt search for a second *GLA* variant.^58^, ^67^ Biopsy to check for sphingolipids deposits may be considered,^34^ although histological confirmation of VUS pathogenicity may be challenging. Indeed, documentation of only a few Gb_3_ inclusions in some cells does not necessarily imply pathogenicity. Electron microscopy can better assess the amount of stored material, although the sampled tissue and the cellular type (terminally‐differentiated vs. others) may yield different results and interpretation should consider phenocopies of FD (e.g., amiodarone intake).

### 5.3 Compare allele frequencies of unknown *GLA* variants with prevalence of FD, and with frequency of the most common pathogenic allele

Traditionally, an AF < 1% supports pathogenicity and >1% benignity. The most frequent pathogenic *GLA* variants (p.(Asn215Ser) in Caucasians and IVS4 + 919G>A in Asians) occur indeed at a frequency <1%. However, this rule does not always apply, as exemplified by FD, where many *GLA* variants considered or proven as benign (p.(Glu66Gln), p.(Arg118Cys), p.(Ser126Gly), p.(Ala143Thr), p.(Asp313Tyr)) do not meet the >1% frequency threshold.

When considering the pathogenicity of a *GLA* variant, if the variant's AF is higher than 0.0125% (FD prevalence: 1/8000) and higher than the frequency of the most common pathogenic allele (indicated as the “Popmax Filtering AF” in GnomAD), the variant should be considered as “likely benign.”^13^, ^66^, ^68^ Furthermore, the variant's AF may vary in different populations and the highest one should be used to determine the likelihood of pathogenicity. Since FD is considered underdiagnosed, ruling out a variant as non‐pathogenic, due to a high AF could be questionable. However, statistical models (http://cardiodb.org/allelefrequencyapp/) can help compute the maximum expected AF and provide better estimates of disease‐causing variants in the general population (maximum credible population AF).^68^

Once pathogenicity of a missense variant has been verified, the next step is to determine its association with a specific phenotype. Whether the “classic” or “later‐onset” phenotype of FD is more prevalent is still unclear; several arguments exist for both hypotheses. The total number of genetic variants responsible for classic FD overwhelms the number of genetic variants associated with later‐onset FD (Figure 2).^69^ However, a few later‐onset variants have been found at relatively higher frequency. Later‐onset phenotype with single‐organ impact and lower morbidity has minimal effects on genetic fitness, with undetected but higher segregation within populations and consequent higher prevalence.^51^ Familial segregation analysis of a *GLA* VUS can be performed for further information. Absence of co‐segregation strongly suggests benignity while presence of co‐segregation is in favor pathogenicity.^70^ However, identifying relatives who can effectively contribute to pathogenicity classification of a VUS, is not always feasible, especially when no male relative exists, thereby limiting the relevance of α‐Gal A assay.^71^

### 5.4 Periodically review the literature and specific databases

It is important to periodically review the literature and perform a specific database search for additional evidence on pathogenicity/benignity of variants, since with growing evidence over time, some variants originally designated VUS may be reclassified as either likely pathogenic/pathogenic or in contrast likely benign/benign and in the latter case, no longer considered to be FD‐causing variants.^35^, ^54^, ^58^, ^59^, ^62^, ^72^ However, careful consideration is necessary when using the variants classification from these databases, as they allow inclusion of different levels of evidence without a hierarchical ranking, such as an in vitro expression study with no clinical data or a report of a few poorly detailed clinical cases^73^ which may bias the final interpretation of pathogenicity of a variant under study.

## 6 CONCLUSION

Since the publication of ACMG‐AMP,^13^ the approach to interpreting unknown/novel *GLA* variants integrates information from in vitro prediction softwares, AF and gene databases.^13^ However, this traditional approach is not universally applicable for rare diseases. We recommend that physicians should be aware of potential errors in interpreting *GLA* variants. Misdiagnosis prolongs patient diagnostic odyssey, resulting in higher morbidity and adding unnecessary financial burden to the healthcare system.^69^ Accurate interpretation benefits from the input of rare disease expert clinicians and geneticists (Table 4; Figure 3). We call for more data sharing by referral centers worldwide to increase the robustness of disease specific databases. These lessons obtained from Fabry disease contribute, with practical concepts, to better interpret the pathogenicity of allelic variants in genetic diseases for a more accurate diagnosis and effective management.

## CONFLICT OF INTEREST

D.P.G.: Consultant for Sanofi‐Genzyme, Idorsia, Takeda; speaker's honoraria from Takeda, Amicus, Sanofi‐Genzyme. T.L.: Hotel/travel grants from Sanofi‐Genzyme, Takeda, BioMarin, Enzyvant, Orphan. E.H.: Speaker's honoraria and/or travel grants from Sanofi‐ Genzyme, GSK, Actelion, Sobi; B.K.: Speaker's honoraria from Travere, Sanofi, Alnylam, Reata; hotel/travel grants from Sanofi, Travere, Reata; D.L.: Speaker's honoraria and hotel/travel expenses from Amicus, Sanofi‐Genzyme. V.L.S.: Speaker's honoraria from Sanofi‐Genzyme; hotel/travel grants from Amicus, Orphan, Takeda, Sanofi‐Genzyme; K.N.: Speaker's honoraria, travel grants from Sanofi Genzyme; grants from Amicus therapeutics; E.N.: Speaker's honoraria and/or travel grants from Amicus Therapeutics, Sanofi‐Genzyme. J.P.R.: Consultant for Sanofi‐Genzyme; hotel/travel expenses from Amicus Therapeutics; speaker's honoraria from Amgen.

## AUTHOR CONTRIBUTIONS

Each author participated in the meetings, analyzing literature, revising, and interpreting the presented cases with current data, establishing recommendations and approved the manuscript. Dominique P. Germain designed and supervised the study, substantially contributed to developing the manuscript and wrote the revised version.

## Data Availability

Data are not shared due to patient confidentiality and ethical restrictions.
